# Supplementary material for: Role and mechanism of NCAPD3 in promoting malignant behaviors in gastric cancer
Source: Front Pharmacol. 2024 Apr 22;15:1341039. doi: 10.3389/fphar.2024.1341039 (PMC11070777; doi:10.3389/fphar.2024.1341039)
Supplement: Supplementary file 11 [file DataSheet2.ZIP › GSEA/GO_CC/my_analysis.Gsea.1599461408869/GO_SYNAPSE.html]

Details for gene set GO\_SYNAPSE[GSEA]

|  || Dataset | filtered\_dataset.sample\_info.cls#WT\_versus\_NCAPD3\_MUT |
| Phenotype | sample\_info.cls#WT\_versus\_NCAPD3\_MUT |
| Upregulated in class | NCAPD3\_MUT |
| GeneSet | GO\_SYNAPSE |
| Enrichment Score (ES) | -0.2332802 |
| Normalized Enrichment Score (NES) | -1.669267 |
| Nominal p-value | 0.020895522 |
| FDR q-value | 0.10806045 |
| FWER p-Value | 0.617 |
Table: GSEA Results Summary

  

Fig 1: Enrichment plot: GO\_SYNAPSE      
 Profile of the Running ES Score & Positions of GeneSet Members on the Rank Ordered List

  

| SYMBOL | TITLE | RANK IN GENE LIST | RANK METRIC SCORE | RUNNING ES | CORE ENRICHMENT || 1 | 659 | BMPR2 | 16 | 1.047 | 0.0103 | No |
| 2 | 2243 | FGA | 18 | 1.039 | 0.0316 | No |
| 3 | 323 | APBB2 | 35 | 0.977 | 0.0404 | No |
| 4 | 9759 | HDAC4 | 46 | 0.925 | 0.0526 | No |
| 5 | 4204 | MECP2 | 70 | 0.856 | 0.0535 | No |
| 6 | 7976 | FZD3 | 88 | 0.815 | 0.0581 | No |
| 7 | 4193 | MDM2 | 131 | 0.769 | 0.0430 | No |
| 8 | 23380 | SRGAP2 | 142 | 0.757 | 0.0515 | No |
| 9 | 7248 | TSC1 | 158 | 0.718 | 0.0556 | No |
| 10 | 134957 | STXBP5 | 170 | 0.709 | 0.0624 | No |
| 11 | 613 | BCR | 180 | 0.695 | 0.0704 | No |
| 12 | 54434 | SSH1 | 183 | 0.692 | 0.0837 | No |
| 13 | 6191 | RPS4X | 196 | 0.682 | 0.0892 | No |
| 14 | 9456 | HOMER1 | 198 | 0.680 | 0.1029 | No |
| 15 | 8831 | SYNGAP1 | 236 | 0.647 | 0.0889 | No |
| 16 | 5701 | PSMC2 | 244 | 0.636 | 0.0972 | No |
| 17 | 5728 | PTEN | 285 | 0.606 | 0.0800 | No |
| 18 | 54477 | PLEKHA5 | 286 | 0.605 | 0.0929 | No |
| 19 | 23229 | ARHGEF9 | 317 | 0.584 | 0.0828 | No |
| 20 | 2244 | FGB | 321 | 0.582 | 0.0930 | No |
| 21 | 8573 | CASK | 368 | 0.554 | 0.0702 | No |
| 22 | 1183 | CLCN4 | 373 | 0.551 | 0.0790 | No |
| 23 | 9743 | ARHGAP32 | 413 | 0.528 | 0.0609 | No |
| 24 | 200933 | FBXO45 | 456 | 0.500 | 0.0401 | No |
| 25 | 6507 | SLC1A3 | 464 | 0.496 | 0.0454 | No |
| 26 | 57460 | PPM1H | 502 | 0.472 | 0.0277 | No |
| 27 | 3184 | HNRNPD | 508 | 0.470 | 0.0339 | No |
| 28 | 104 | ADARB1 | 518 | 0.463 | 0.0370 | No |
| 29 | 1979 | EIF4EBP2 | 532 | 0.456 | 0.0369 | No |
| 30 | 3836 | KPNA1 | 548 | 0.448 | 0.0352 | No |
| 31 | 6208 | RPS14 | 604 | 0.421 | 0.0029 | No |
| 32 | 10280 | SIGMAR1 | 655 | 0.399 | -0.0261 | No |
| 33 | 57679 | ALS2 | 656 | 0.399 | -0.0176 | No |
| 34 | 26747 | NUFIP1 | 666 | 0.394 | -0.0160 | No |
| 35 | 54476 | RNF216 | 672 | 0.389 | -0.0115 | No |
| 36 | 2932 | GSK3B | 714 | 0.367 | -0.0344 | No |
| 37 | 3188 | HNRNPH2 | 769 | 0.338 | -0.0677 | No |
| 38 | 23767 | FLRT3 | 781 | 0.333 | -0.0689 | No |
| 39 | 1605 | DAG1 | 823 | 0.304 | -0.0932 | No |
| 40 | 3312 | HSPA8 | 848 | 0.244 | -0.1060 | No |
| 41 | 3673 | ITGA2 | 858 | -0.264 | -0.1071 | No |
| 42 | 55450 | CAMK2N1 | 861 | -0.269 | -0.1029 | No |
| 43 | 9181 | ARHGEF2 | 902 | -0.326 | -0.1260 | No |
| 44 | 301 | ANXA1 | 914 | -0.342 | -0.1270 | No |
| 45 | 3913 | LAMB2 | 919 | -0.348 | -0.1226 | No |
| 46 | 1978 | EIF4EBP1 | 938 | -0.362 | -0.1284 | No |
| 47 | 5621 | PRNP | 970 | -0.387 | -0.1434 | No |
| 48 | 9572 | NR1D1 | 977 | -0.392 | -0.1396 | No |
| 49 | 80315 | CPEB4 | 1053 | -0.448 | -0.1863 | No |
| 50 | 1191 | CLU | 1056 | -0.450 | -0.1782 | No |
| 51 | 5818 | PVRL1 | 1073 | -0.458 | -0.1805 | No |
| 52 | 3556 | IL1RAP | 1099 | -0.476 | -0.1891 | No |
| 53 | 10681 | GNB5 | 1105 | -0.481 | -0.1826 | No |
| 54 | 7074 | TIAM1 | 1143 | -0.508 | -0.1996 | No |
| 55 | 3706 | ITPKA | 1150 | -0.514 | -0.1931 | No |
| 56 | 2119 | ETV5 | 1176 | -0.545 | -0.2003 | No |
| 57 | 153 | ADRB1 | 1221 | -0.595 | -0.2206 | Yes |
| 58 | 8061 | FOSL1 | 1222 | -0.597 | -0.2079 | Yes |
| 59 | 2043 | EPHA4 | 1228 | -0.602 | -0.1989 | Yes |
| 60 | 22931 | RAB18 | 1231 | -0.608 | -0.1874 | Yes |
| 61 | 26999 | CYFIP2 | 1234 | -0.611 | -0.1759 | Yes |
| 62 | 1956 | EGFR | 1266 | -0.657 | -0.1852 | Yes |
| 63 | 286 | ANK1 | 1284 | -0.672 | -0.1837 | Yes |
| 64 | 64854 | USP46 | 1300 | -0.702 | -0.1800 | Yes |
| 65 | 54464 | XRN1 | 1301 | -0.703 | -0.1650 | Yes |
| 66 | 885 | CCK | 1302 | -0.703 | -0.1501 | Yes |
| 67 | 57348 | TTYH1 | 1306 | -0.710 | -0.1372 | Yes |
| 68 | 3084 | NRG1 | 1320 | -0.739 | -0.1313 | Yes |
| 69 | 6509 | SLC1A4 | 1330 | -0.752 | -0.1220 | Yes |
| 70 | 7779 | SLC30A1 | 1332 | -0.755 | -0.1067 | Yes |
| 71 | 10611 | PDLIM5 | 1335 | -0.758 | -0.0921 | Yes |
| 72 | 4734 | NEDD4 | 1369 | -0.856 | -0.0986 | Yes |
| 73 | 9990 | SLC12A6 | 1379 | -0.922 | -0.0858 | Yes |
| 74 | 10397 | NDRG1 | 1380 | -0.925 | -0.0661 | Yes |
| 75 | 6711 | SPTBN1 | 1381 | -0.925 | -0.0464 | Yes |
| 76 | 4919 | ROR1 | 1385 | -0.954 | -0.0284 | Yes |
| 77 | 8867 | SYNJ1 | 1387 | -0.979 | -0.0083 | Yes |
| 78 | 493 | ATP2B4 | 1401 | -1.166 | 0.0068 | Yes |
Table: GSEA details [plain text format]

  

Fig 2: GO\_SYNAPSE      
 Blue-Pink O' Gram in the Space of the Analyzed GeneSet

  

Fig 3: GO\_SYNAPSE: Random ES distribution      
 Gene set null distribution of ES for **GO\_SYNAPSE**
